# Spectroscopic analyses for possible transport of polyaromatic hydrocarbons onto fish in Mediterranean Sea

**DOI:** 10.1038/s41598-025-98472-4

**Published:** 2025-09-30

**Authors:** Noha M. Sabry, Samah M. Bassem, Tarek A. Temraz, Fagr Kh. Abdel-Gawad, Ahmed Refaat, Hanan Elhaes, Medhat A. Ibrahim

**Affiliations:** 1 Water Pollution Research Department, Environment and Climate Change Research Institute, Centre of Excellence for Research and Applied Studies on Climate Change and Sustainable Development (C3SD-NRC), National Research Centre, 33 El-Bohouth St., Dokki, Giza, 12622 Egypt; 2 Department of Marine Science, Faculty of Science, Suez Canal University, Ismailia, Egypt; 3 Spectroscopy Department, National Research Centre, 33 El-Bohouth St., Dokki, Giza, 12622 Egypt; 4 Molecular Modeling and Spectroscopy Laboratory, Centre of Excellence for Advanced Science, National Research Centre, 33 El-Bohouth St., Dokki, Giza, 12622 Egypt; 5 Physics Department, Faculty of Women for Arts, Science and Education, Ain Shams University, Cairo, 11757 Egypt

**Keywords:** DFT:B3LYP/6-31G(d,p), FTIR, Tilapia fish, Bacterial quality, Genotoxicity, Environmental sciences, Materials science

## Abstract

Metal pollution in the Mediterranean coast is a growing environmental concern. The effect of trace metals and polycyclic aromatic hydrocarbons (PAHs) on fish was subjected to DFT:B3LYP/6-31G(d,p) computational study. Both alanine (Ala) and phenylalanine (PAla) were used as model molecules for protein of fish. The interaction of Ala and PAla with metals resulted in significant decrease of the HOMO–LUMO bandgap energy of Ala and PAla from 2.6647 and 1.5772 eV, down to 1.2871 and 1.2675 eV, respectively, reflecting increased reactivity for further interaction with the surrounding environment. Results also indicated that interaction of both metals and PAHs with protein resulted in changing the structure of the amide groups with significant shift in their band positions. Changes in the geometrical parameters of protein were detected which, in turn, changed the amount of energy required to vibrate its funcitonal groups, thus leading to a change in the vibrational features of COOH and NH_2_. FTIR spectra of fish gills, liver, and muscle tissues collected from four sites in Alexandria (El-Shatby, Qaitbay, Al Asafra, and El-Max) confirmed the computational findings, revealing alterations in protein secondary structures. Experimental studies further assessed the effects of pollutants on fish health. Bacterial analysis showed the highest levels of *Staphylococcus aureus* (1.5 × 10^4^ CFU/ml) and *Escherichia coli* (5.0 × 10^3^ CFU/g) in El-Max, while the lowest bacterial counts were recorded in El-Shatby. Micronucleus analysis indicated significant genotoxic effects, with higher micronuclei frequencies in fish from El-Max than in El-Shatby. Gene expression analysis revealed that fish from El-Max exhibited upregulated levels of Cytochrome c, P53, and TNF genes, suggesting oxidative stress and apoptosis as potential responses to environmental pollution. One-way ANOVA confirmed significant differences between control and polluted groups (*p* < 0.05), with the highest expression levels observed in Tilapia liver samples. These findings highlight the detrimental impact of PAHs and heavy metals on aquatic organisms, emphasizing the need for continuous environmental monitoring and pollution control measures in the Mediterranean coastal waters.

## Introduction

The Mediterranean Sea is rich in its biodiversity and considered a hot spot for some fish species. Many factors contribute to cause the biodiversity loss such as; habitat loss, overexploitation, pollution and climate change^1,2^. The coastal zones suffer from enormous pollutants coming from various sources; industrial, agricultural and mining procedures affecting the marine environment^3^. Coastal ecosystems act as primary element of conservation of coastal zones and significantly impact social progress, population extension and marine environment^4,5^. Climate change, sea-level rise and anthropogenic activities, such as; coastal improvement, aquaculture, agriculture and industry all cause changes in such coastal areas ecosystems^6,7^.Besides AHCs, the marine environment is also subjected to heavy metals pollution, which attracted attention because they are non-degradable, remain and have serious impacts especially at high level^8–10^. Although some metals are essential for biological operations and the sustainability of surrounding ecosystem, other metals cause dangerous effect even at low levels^11^. Sediments act as the main store of heavy metals in the marine environment having notable impact in storage and transport of potentially toxic metals that can be hazardous to marine organisms^12^. It was also stated that, heavy metals could be absorbed and bio-accumulated by fish leading to hazardous impacts to seafood consumers which needs application of continuous monitoring to ensure suitable environmental management of fish and seafood products. Moreover, fish was always used as bio indicator of different contaminants including toxic metals to evaluate water quality in marine environment^13–15^. Monitoring of water quality is an important strategy for evaluating biological and physical characteristics for early prediction of pollution sources which in turn aid in supporting the overall management of coastal zones^16,17^. Heavy metals in the environment are of course of great importance according to their impact. But there are other sources of pollution such as organic pollution. Polycyclic aromatic hydrocarbons (PAHs), is a class of chemical compounds with two or more condensed aromatic rings, PAHs is frequently ubiquitous compounds in the environment^18–20^. PAHs finds its ways to the aquatic environment throughout industrial and petroleum activities then became available to aquatic organisms, such as fish, through feeding and absorption via gills, this in turn leads to possible of bioaccumulation and consequently trophic biomagnification^21^. The occurrence of PAHs in the aquatic environment leads to four main types derived from fuels (petrogenic), derived from an incomplete combustion process (pyrogenic), generated by organic metabolism (biogenic), and generated by the transformation process in sediment (diagenetic)^22^. Generally, hydrocarbon is considered the major components of crude oil and is classified among the PAHs, including aliphatic saturated hydrocarbons, aliphatic unsaturated hydrocarbons, and alicyclic saturated hydrocarbons^23^. The impact of PAHs on the ecosystem is regarded concerning its specific toxicity^24^. It is stated that, oil spill accidents contained PAHs that had toxic effects, such as immunotoxicity, embryonic abnormalities, and cardiotoxicity, for wildlife including fish, benthic organisms, and marine vertebrates^25,26^. This makes the PAHs carcinogenic chemicals and classified among the important organic pollutants affecting both the environment and human society^27^. In this sense, the PAHs are transported into cells according to their hydrophobicity, which induces gene expression of the cytochrome P450 (CYP) enzyme group^28^. It is stated that, the expressed CYP enzymes metabolize PAHs into additional metabolites. This makes that, it is important to note that several intermediates in this metabolic pathway can bind to DNA and become mutagenic/carcinogenic^29^. Among many organic pollutants the PAHs is classified of important environmental concern according to its mutagenic, carcinogenic, endocrine disrupting and reproductive toxicity^30^. As a result of the direct effect of PAHs on fish, fish becoming a biological indicator for PAHs contamination^31^. It is regarded that, fish as well as other aquatic organism tends to accumulate PAH and in event predisposes the consumer of aquatic foods to certain health risks^32^. Possible accumulation of PAHs as well as other pollutants in fish and other forms of aquatic organism leads to significant health risk to humans^33^. A fraction of pollutants in water may be finds its ways to aquatic organism while other part could be sink into sediment^34^. The problem of pollutants in sediment may be raised as the pollutant is remobilized once more to water with other forms which in turn leads to possible adverse impact of the aquatic environment^35^. One can reveal that, continuous monitoring of the fate and transport of pollutants such as PAHs in sediment, water and fish is an important point of research in both experimental and modeling scale to assign the possible route to control them.

Gathering this knowledge together, it is clear that both PAHs and heavy metals find their ways to both sediment and fish. These may be a hot point of research to elucidate the fate of them in the ecosystem. Molecular modeling is a class of compactions used to elucidate important parameters for molecular systems. It could be an interesting tool for elucidating physical, chemical parameters especially in the field of environment. Molecular modeling applications in the environmental filed showed an interesting result. It was applied to predicate the fate of dioxin in the environment^36^. Later on, the effect of dioxin upon the protein of fish was elucidated by molecular modeling^37^. Results were verified with experimental tools indicating that protein is diversely affected by dioxin in the aquatic environment. It was also used to investigate the structural changes in protein under the influence of heavy metals. It was stated that, protein structure is affected by the presence of heavy metals^38^. Modeling was used to design and implement microsphere from aquatic plant to remediate heavy metals such as Pb and Cd from wastewater^39,40^. Results indicated that beside the unique surface of the microsphere some bonding responsible for coordinating metals and enhancing the process of metal uptake by the studied microspheres.

The present work is conducted to model the effect of trace metals and PAHs on the fish using DFT:B3LYP/6-31G(d,p) level. Water, sediment, and fish covering some sites in Alexandria were collected to verify the model. Water quality, ICP survey for inorganic pollutants beside FTIR molecular structure was determined.

## Materials and methods

### Calculations details

**Fig. 1:**
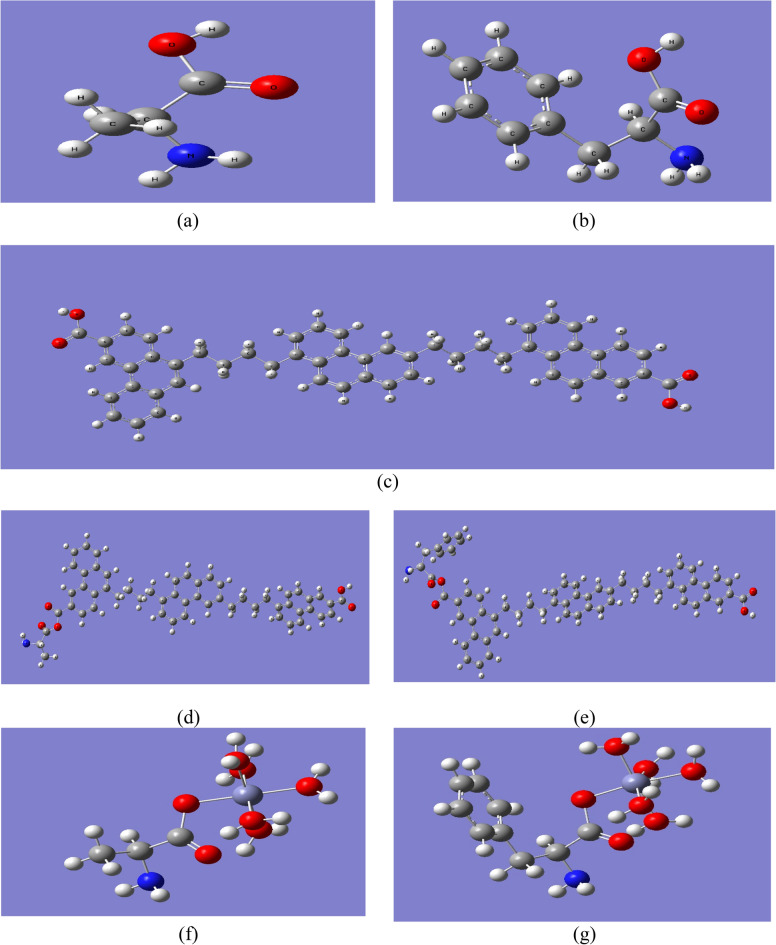
The studied model molecules of (**a**) Amino acid Ala; (**b**) Amino acid PAla; (**c**) Proposed PAHs model molecule; (**d**) Proposed PAHs model molecule interacted with Ala; (**e**) Proposed PAHs model molecule interacted with PAla; (**f**) Amino acid Ala interacted with hydrated Zn; and (**g**) Amino acid PAla interacted with hydrated Zn. These models were generated with Gauss view 6.0 in G09 Softcode.

Gauss view 6.0 was used to build the model molecules in the present work. Both alanine (Ala) and phenylalanine (PAla) are proposed to simulate the structure of proteins. Ala is an indication for protein in fish gills and liver indicated in Fig. 1a. PAla is simulating the protein in fish muscle is indicated in Fig. 1b. A model for PAHs is constructed as indicated in Fig. 1c, then abbreviated as PAHs. This model is further supposed to interact with amino acid Alaas indicated in Fig. 1d, forming PAHs-Ala which simulates the interaction between PAHs and protein in both gills and livers. PAHs is then supposed to interact with PAla as indicated in Fig. 1e, forming PAHs-PAla. The amino acid Alais further interacted with hydrated Zn as indicated in Fig. 1f. Finally, the amino acid PAla is supposed to be interacted with hydrated Zn as indicated in Fig. 1g.

All the studied model molecules were subjected to optimization using Gaussian program^41^ at Molecular Modeling and Spectroscopy Laboratory, Centre of Excellence for Advanced Science, National Research Centre, Egypt. Optimization was conducted with Becke style -three parameters-Lee Yang-Parr hybrid functional (B3LYP)^42–44^ using 6-31g(d,p) basis set. Some parameters were calculated also with DFT to evaluate the reactivity of the studied structures which included electron affinity (EA =  − E_LUMO_), ionization potential (IP =  − E_HOMO_), global hardness (η = (IP − EA)/2), chemical softness (σ = 1/η), electronegativity (χ = (IP + EA)/2), and electrophilicity index (ω)^45,46^. Chemical hardness and softness were also calculated. These parameters simulate the chemical reactivity and stability of structures.

### Sampling

#### Collection of water and sediment samples

**Fig. 2:**
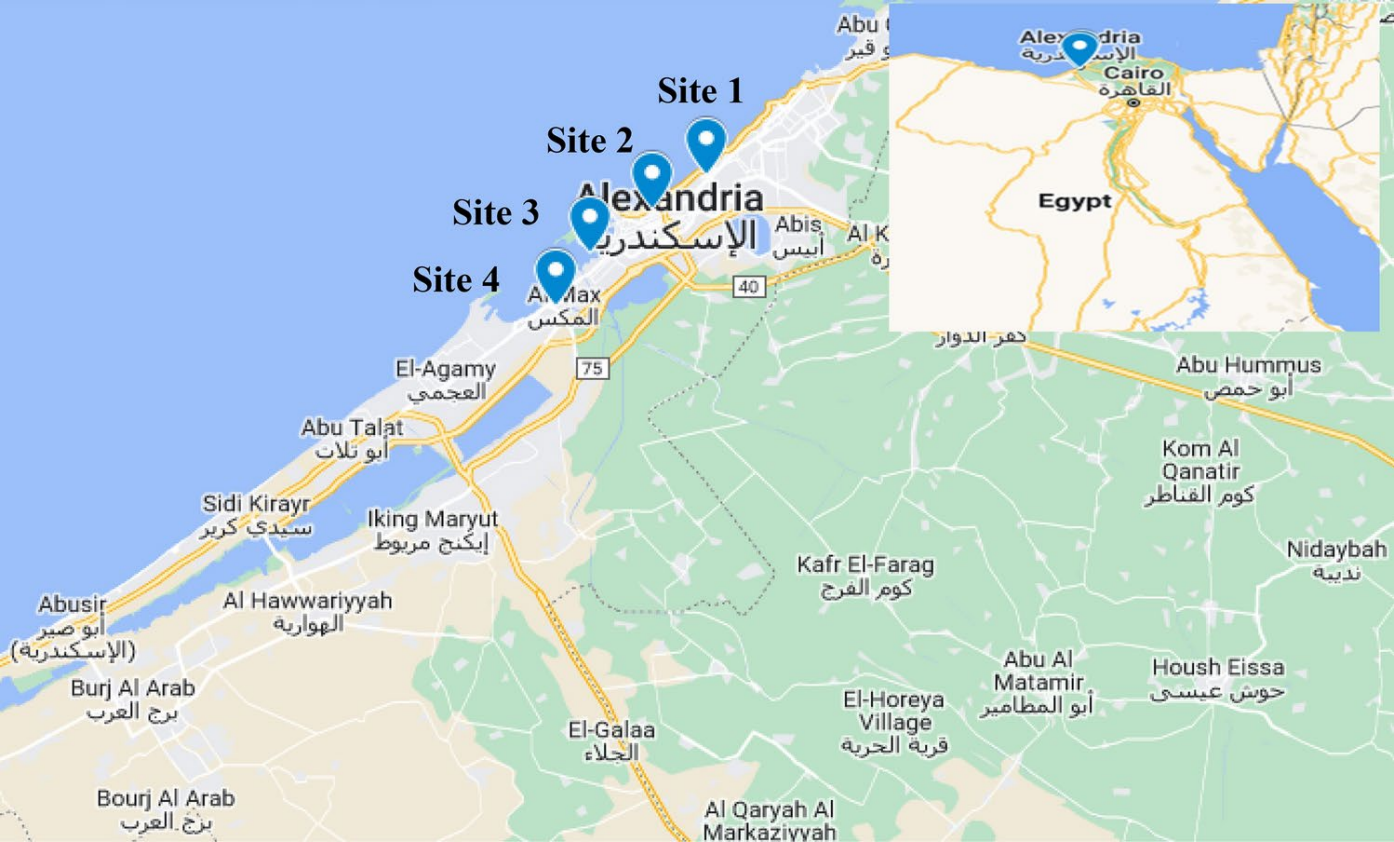
Map of Alexandria location and different sampling sites (n = 4), this map is generated from Google Maps screenshot, according to Google’s guidelines, such images can be used in research papers with proper attribution.

Sampling was done at four sites representing different pollution levels in Alexandria city-Egypt, which were determined by Geographic Position System (GPS) as shown in (Table 1 and Fig. 2).

**Table 1:** Sampling sites at different pollution levels in Alexandria.

Sites	Coordination	
	Latitude / Longitude	
Site 1: El-Shatby	31º 12′ 42″ N	29º 54′ 54″ E
Site 2: Qaitbay Citadel	31º 12′ 32″ N	29º 52′ 57″ E
Site 3: Al Asafra	31º 16′ 22″ N	29º 59′ 59″ E
Site 4: El-Max	31º 9′ 8'' N	29º 50′ 33″ E

Water samples were collected in Nansen bottles crafted from Teflon to ensure inertness with all measured parameters. Sediment samples were also obtained using a grab sampler provided by Hydro-bios, Kiel, Germany, to encompass a comprehensive analysis of the area’s environmental condition.

#### Collection of fish samples

Some important fresh fish was randomly and periodically collected from different sites along Alexandria governorate, Egypt (2023–2024), Table 1. The samples were kept in separate plastic bags and transferred directly without undue delay to the laboratory in an insulating refrigerated container under complete aseptic conditions to avoid any changes in the quality of the sample. Samples were examined bacteriologically immediately after arrival to the laboratory for isolation of some pathogenic bacteria. The samples were prepared according to International Organization of Standardization ISO 4833–1:2013^47^. Fish samples were dissected to isolate liver for genetic experiments and preserved in − 80 ℃.

### Instrumentation

#### ICP measurements for heavy metals

Determination of metal ions was conducted using the Agilent 5100 Synchronous Vertical Dual View (SVDV) ICP-OES, with Agilent Vapor Generation Accessory VGA 77. All samples were digested to have acceptable matrix for measuring of metals and to provide acceptable and consistent recovery compatible with the analytical method (APHA, 2023^48^). For each series of measurements intensity calibration curve was constructed composed of a blank and three or more standards from Merck Company (Germany). Accuracy and precision of the metal ions measurements were confirmed using external reference standards from Merck, and standard reference material and quality control sample from National Institute of Standards and Technology (NIST), were used to confirm the instrument reading. All the studied samples show concentrations below the limits. As noticed by ICP, the level of the heavy metals is below the detection limits; in this sense one divalent metal is chosen to indicate its effect upon the model molecule. This why the hydrated Zn is chosen to simulate the effect of trace metals upon protein model molecule.

#### Fourier transform infrared spectroscopy

Attenuated total reflection ATR-FTIR spectra were obtained using Vertex 80 FTIR spectrometer from Bruker Optics GmbH, Germany, equipped with diamond ATR crystal system in the spectral range of 4000–400 cm^−1^ with the resolution of 4 cm^−1^. Measurements were conducted at National Research Centre, Egypt.

### Bacteriological examination

Liver and gills were removed then the skin was sterilized by alcohol. The muscles above the lateral line was cut, from which 25 g were taken under aseptic to 225 ml of 0.1% sterile peptone water and homogenized at 1400 rpm for 2.5 min^49^.

#### Determination of Escherichia coli

Isolation of *E. coli* was conducted according to ISO 16,649^50^. Approximately 1 g of homogenized fish fillets was mixed with 9 mL of modified Tryptone Soya Broth (mTSB, HiMedia). The samples were thoroughly mixed and left to incubate overnight at a temperature of 41 °C. Following selective enrichment, 50 µL of the resulting mixture were spread onto MacConkey agar (HiMedia) plates to isolate *E. coli* bacteria, and the plates were incubated aerobically at 37 °C for 24h. The plates were then examined for the presence of *E. coli* growth, characterized by pink colonies indicating lactose fermentation. A single, isolated colony exhibiting these characteristics was chosen and transferred to Eosin Methylene Blue agar (EMB, HiMedia) to observe the formation of a metallic sheen. At the same time, another colony displaying similar characteristics was subjected to gram staining according to ISO 16,649^50^.

#### *Determination of Salmonella *spp*.*

Briefly, 225 mL of buffered peptone water was inoculated with 25 g fish and incubated at 37 °C for 18 h (pre-enrichment in non-selective liquid medium), then inoculate 1 mL of the above-mentioned broth into 9 mL Rappaport–Vassiliadis medium with soya (RVS broth, Oxoid) and finally plating out on Xylose Lysine Deoxycholate agar (XLD, Oxoid) and Salmonella Shigella agar (SS, Oxoid), incubated at 37 °C for 24 h according to ISO 6579^51^.

#### Determination of Staphylococcus aureus

Accurately, 0.1 ml from nutrient broth was spread onto plates of Baird Parker (Oxoid). Plates were incubated at 37 ºC for 48 h. *Staphylococcus* appears as black shiny colonies with narrow white margins surrounded by a clear halo zone extending into the opaque medium^52^.

### Micronucleus analysis

For the analysis of micronucleus, fish blood sample was spread on a clean slide from fish collected from the 4 sites. Then Slides were fixed with methanol after dry at ambient temperature for a whole night. Using an Olympus epifluorescent microscope, blood smears were stained with 0.01 percent Giemsa dye, and 2,000 cells/fish were counted. For every 1,000 cells, the number of micronuclei (MN) was counted^53^.

### Gene expression

#### Isolation of RNA and reverse transcription (RT) reaction

RNA was isolated from collected fish liver tissues using the usual TRIzol® Reagent extraction procedure (Invitrogen, Germany). RNA was hydrolyzed in diethylpyrocarbonate (DEPC) water before use^54^. Then, the RevertAidTM First Strand cDNA Synthesis Kit was used, the entire Poly (A) + RNA extracted from liver tissue was reverse transcribed in a final amount of 20 µl. (MBI Fermentas, Germany).

#### Real time-polymerase chain reaction (RT-PCR)

The Applied Biosystems StepOneTM Real-Time PCR System from Thermo Fisher Scientific, Waltham, MA, USA, was used. Table 2 displays a list of the primer sequences for the employed genes. To evaluate the quality of the employed primers, a melting curve analysis was done at 95.0°C at the conclusion of each qPCR^55^.

**Table 2:** The list of quantitative real-time PCR assays used in this study.

Fish type/ Gene name	Primers F: forward, R: reverse	GenBank Access. No., (References)
Tilapia fish Cyt c	F:caggaacctcaggctgaaga R: cggatcgctctctctccatt	NM_001279575
Tilapia Fish P53	F: aagaacagggcgtggaaaac R: ccggtatccagggtgctaat	GU594898
Tilapia Fish TNF-α	F: cattgtgtcgcctagtctgc R: atggagtcgggtttggagag	XM_013266975
Seabream Fish Cyt c	F: cctgtcctggtctgtgatgt R:atggtgaagggcaggaatga	EU107275
Seabream Fish P53	F: taccatgaacagcagctcca R:gcctcctccttttcctctgt	XM_030443965
Seabream Fish TNF	F: ttggtattggaacggcaagc R:ttctcagcgtggtccttctt	AJ413189.2
Grouper Fish Cyt c	F: gtcccgctaaaccgatcaac R: tgatcctcactgcacaccat	XM_049593510
Grouper Fish P53	F: accatcctgctgagcttcat R: tcctcagttttgcgatccct	HM622380.1
Grouper Fish TNF	F: cgctggtccaaatcatacgg R:ggttgaacacagctcccatg	HQ011926

#### Statistical analysis

The means ± SD are used to represent all of the results from the bacterial analysis parameters and micronucleus analysis. A one-way analysis of variance (ANOVA) was performed to compare gene expression levels among the different sample groups. A significance level of *p* < 0.05 was considered statistically significant, indicating meaningful differences between the groups.

## Results and discussion

### Molecular modeling

**Fig. 3:**
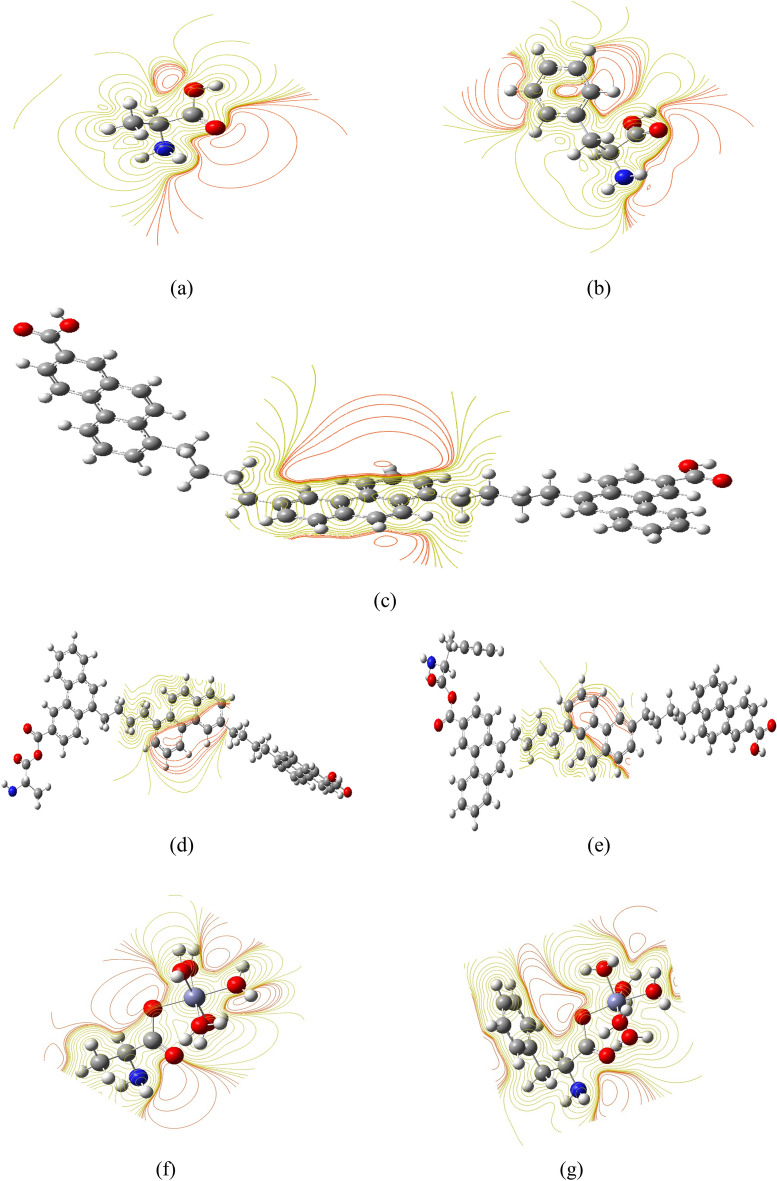
MESP maps for the studied model molecules (**a**) Amino acid Ala; (**b**) Amino acid PAla; (**c**) Proposed PAHs model molecule; (**d**) Proposed PAHs model molecule interacted with Ala; (**e**) Proposed PAHs model molecule interacted with PAla; (**f**) Amino acid Ala interacted with hydrated Zn; and (**g**) Amino acid PAla interacted with hydrated Zn. These models were generated with G09 Softcode and represented with Gauss view 6.0.

The model molecules which are presented in Fig. 1 were optimized with B3LYP/6-31g(d,p) quantum level. Molecular electrostatic potential MESP is mapped as indicated in Fig. 3. As indicated in Fig. 3a. the MESP of the amino acid Ala, the red circles indicated negative potential around both amide and carboxyl group. The same was also regarded for the MESP of the amino acid PAla, in Fig. 3b. The MESP of the PAHs model molecule PAHs is indicated in Fig. 3c. the MESP of the PAHs indicated active site in the ring in the middle of the structure not in the terminals as in case of the amino acids. MESP is one of the important descriptors for reactivity as it indicated the active sites upon the studied surface^46^.

The MESP maps presented in Fig. 3d and e are for the proposed PAHs model molecule, and PAHs interacted with Ala and PAla. The same behavior is indicated whereas the active site remains the middle of the PAHs.

Table 3 presented the calculated total dipole moment TDM (Debye) and HOMO–LUMO bandgap energy ΔE (eV) using DFT: B3LYP/6-31g(d,p) for the studied model molecules. TDM was 6.6404 Debye and 6.2151 Debye for Ala and Pala while ΔEwere 2.6647 eV and 1.5772 eV. It was stated earlier that, higher TDM values with lower HOMO/LUMO is a physical indication for the reactivity of the given structure^38,40^. Applying this concept to the tabulated data in Table 1, high reactivity of the amino acids is recorded.

**Table 3:** Calculated TDM (Debye) and HOMO–LUMO bandgap energy ΔE (eV) using DFT:B3LYP/6-31g(d,p) for the studied model molecules.

Structures	TDM (Debye)	∆E (eV)
Ala	6.6404	2.6647
Pala	6.2151	1.5772
PAHs	4.0662	1.5940
PAHs/Ala	3.8101	2.6772
PAHs/Pala	3.8366	2.5547
Ala/Zn.5H_2_O	1.8576	1.2871
PAla/Zn.5H_2_O	1.8729	1.2675

As compared with amino acids the PAHs show lower TDM 4.0662 Debye with a bit lower ΔE 1.5772 eV, this is also an indication for the reactivity of the PAHs model molecule.

As far as PAHs interacted with both amino acids then TDM became 3.8101 Debye and 3.8366 Debye (for PAHs/Ala & PAHs/Pala) while ΔE increased to be 2.6772 eV and 2.5547 eV. This may be an indication that amino acids as a model molecule for protein interacting with PAHs forming stable structure, the stability is noticed for two types of proteins in terms the two proposed model molecules for proteins.

Table 4 presented the DFT: B3LYP/6-31g(d,p) calculation for global reactivity descriptors such as the ionization potential (I), electronic affinity (A), electronic chemical potential (μ), chemical hardness (η), absolute softness (S) and electrophilicity index (ω) for the studied structures.The most imporatnt notice her is that, all the studied descriptors remain almost unchanged for PAHs interacted with Ala and Pala, this confirms the previous findings that, protein model molecules interacted with PAHs forming stable structures.

**Table 4:** DFT: B3LYP/6-31g(d,p) calculation for global reactivity descriptors Ionization Potential (I), Electronic Affinity (A), Electronic chemical potential (μ), Chemical hardness (η), Absolute softness (S) and Electrophilicity index (ω)for the studied structures.

Structures	I	A	μ	η	S	ω
Ala	− 0.0063	0.2377	0.1157	− 0.1220	− 8.1957	− 0.0549
PAla	0.2328	0.0044	0.1186	0.1142	8.7566	0.0616
PAHs	0.2086	0.0591	0.1338	0.0747	13.3842	0.1199
PAHs/Ala	0.2096	0.0696	0.1396	0.0700	14.2837	0.1392
PAHs/PAla	0.2096	0.0686	0.1391	0.0705	14.1854	0.1373
Ala/Zn.5H_2_O	0.0003	0.0476	0.0239	− 0.0237	− 42.2833	− 0.0121
PAla//Zn.5H_2_O	− 0.0028	0.0105	0.0038	− 0.0067	− 150.2630	− 0.0011

### Effect of pollutants on protein structure of fish

To model the effect of pollutants on protein structure of fish, a model protein of Ala and/or PAla were used as model molecules for protein as they are common amino acids found in the protein of fish.

The effect of pollutants was studied upon the vibrational and structural changes upon amino acid of protein model molecules.

Table 5 presented the vibrational assignment for the IR frequencies for amino acid and amino acid interacted with hydrated Zn and/or PAHs which calculated with B3LYP/6-31g(d,p).

**Table 5:** Vibrational assignment for the IR frequencies for amino acid and amino acid interacted with hydrated Zn and/or PAHs which calculated with B3LYP/6-31g(d,p).

Ala	Ala/Zn.5H_2_O	PAla	PAla/Zn.5H_2_O	Band assignment
1647.84	1608.28	1648.86	1612.55	NH_2_scissoring
1845.11	1688.45	1836.48	1740.75	C=O stretching

Ala	Ala/PAHs	PAla	PAla/PAHs	Band assignment
1647.84	1682.77	1648.86	1675.13	NH_2_scissoring
1845.11	1882.86	1836.48	1874.36	C=O stretching

As indicated in Table 5, for Ala the NH_2_scissoring band is located at 1647.84 cm^−1^, then C=O stretching 1845.11 cm^−1^. Ala is interacted with Zn.5H_2_O then the same bands are shifted to lower wavenumber1608.28 cm^−1^ for NH_2_scissoring and 1688.45 cm^−1^ for C=O stretching. As Ala is interacted with PAHs the studied bands are shifted to higher wavenumber 1682.77 cm^−1^ and 1882.86 cm^−1^, respectively.

Table 6 presented the calculated structural parameters for amino acid and amino acid interacted with hydrated Zn and/or PAHs, calculated with B3LYP/6-31g(d,p). Structural parameters are indicated in terms the bond distances for COOH group namely L_C–O_, L_C=O_ beside the OCO angle. For amid group the distances L_N–H_, L_N–H_ beside the HNH angle.

**Table 6:** Structural parameters for amino acid and amino acid interacted with hydrated Zn and/or PAHs calculated with B3LYP/6-31g(d,p).

Ala	Ala/Zn.5H_2_O	PAla	PAla/Zn.5H_2_O	Structural parameters
1.3524	1.5038	1.3464	1.2931	L_C–O_
1.2117	1.2027	1.2136	1.2460	L_C=O_
122.897	126.461	123.198	125.817	OCO
1.0162	1.000	1.0171	1.0153	N–H
1.0178	1.000	1.0183	1.0172	N–H
107.821	109.471	107.374	108.224	HNH

Ala	Ala/PAHs	PAla	PAla/PAHs	Structural parameters
1.3524	1.3916	1.3464	1.3805	L_C–O_
1.2117	1.2003	1.2136	1.2036	L_C=O_
122.897	123.449	123.198	124.250	COO
1.0162	1.0179	1.0171	1.0180	N–H
1.0178	1.0190	1.0183	1.0194	N–H
107.821	104.922	107.374	105.908	HNH

From the table the geometry of COOH is changed as a result of interaction between Ala and Zn forming Ala/Zn.5H_2_O, the same took place as Zn interacted with PAla forming PAla/Zn.5H_2_O.

Regarding the geometry of NH_2_, the structural parameters are changed as Zn and/or PAHs interacted with both Ala and Pala.Correalting the effect of pollutants on both vibrational and structural parameters, one can reveal that as far as the bond distances and bond angles are changed, the energy required to vibrate the protein is changed which, in turn, leads to a change in the main features of COOH and NH_2_.

### Molecular structure and protein secondary structures

**Fig. 4:**
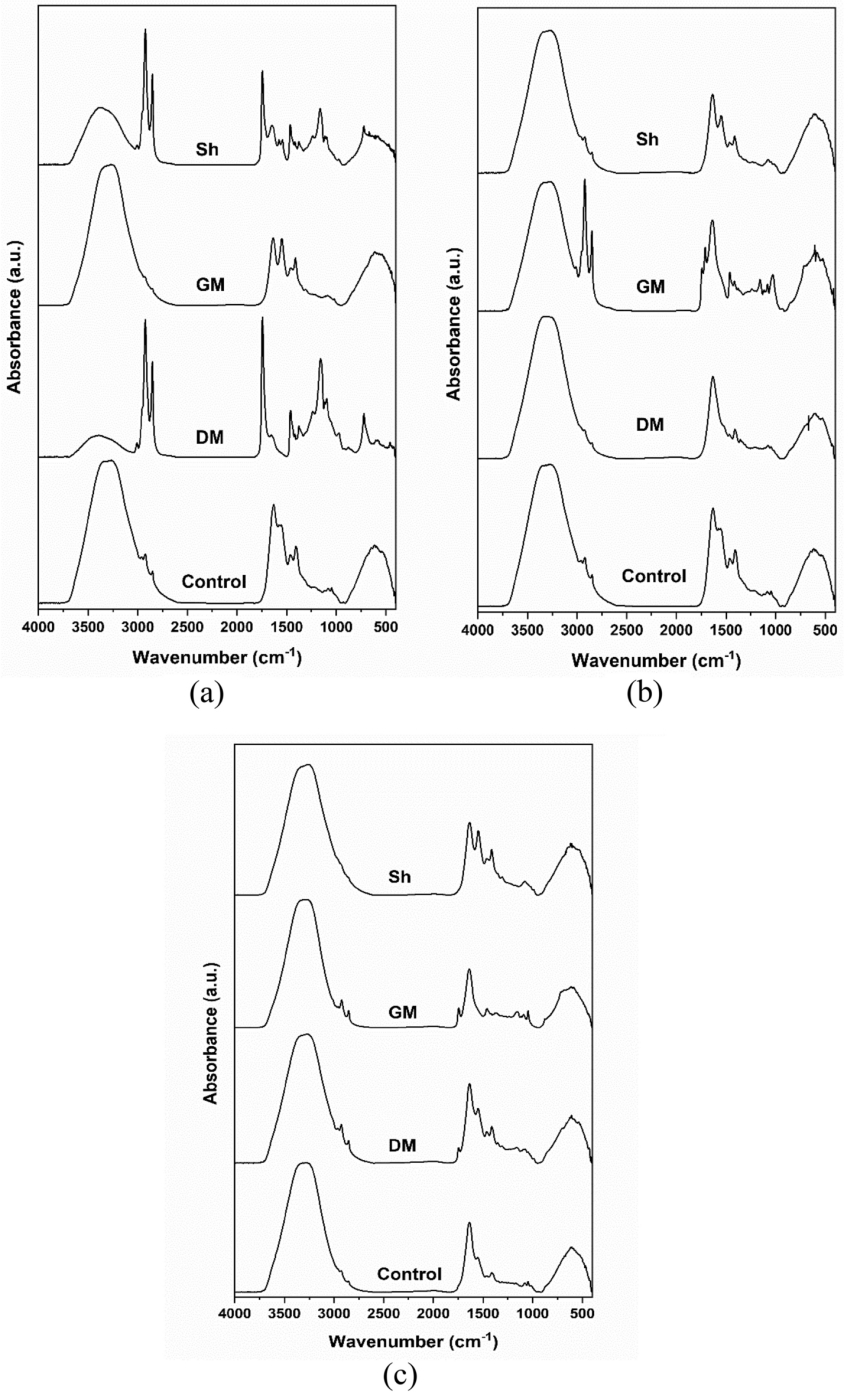
FTIR spectra of (**a**) gills; (**b**) liver; and (**c**) muscles of control and polluted fish samples.

Figure 4a, b and c demonstrate the FTIR spectra of gills, liver and muscles, respectively, of control and polluted fish samples. The detailed band assignments^56,57^ of control samples are shown in Table 7.

**Table 7:** Band assignments of the FTIR spectra of gills, liver and muscle control samples.

Wavenumber (cm^−1^)			Assignment
Gills	Liver	Muscles	
3279	3286	3283	O–H coupled with N–H stretching vibrations
–	2961	–	CH_3_ asymmetric stretching
2926	2923	2928	CH_2_ asymmetric stretching
2853	2853	2855	CH_2_ symmetric stretching
1635	1630	1635	Amide I C=O of proteins
1550	1556	1553	Amide II N–H bend with C–N of proteins
1461	1462	1461	CH_2_ Asymmetric bending
1406	1405	1411	CH_2_ wagging
1080	1082	1078	C–O stretching vibration in collagen and Symmetrical vibration of phosphodiester bond
1045	1045	1045	C–O stretching vibration of carbohydrate residues in collagen

The FTIR spectra of the gills of polluted fish samples demonstrated obvious differences in the spectral features when compared with the control. The amide I band is conformationally sensitive and is affected by changes in hydrogen bonding^58^. Increased intermolecular hydrogen bondings result in shift of amide I bands to lower wavenumbers (redshift), while disruption of the intermolecular hydrogen bondings results in blueshift, whereas denaturation of protein results in decreased intensity of amide I band^59^. As seen in Fig. 4a, amide I band demonstrated significant shift of its position to higher wavenumbers up to 1656 and 1649 cm^−1^ as well as decrease in its intensity in the spectra of DM and Sh, respectively, which may be attributed to disruption of the intermolecular hydrogen bonds^60,61^ associated with denaturation of protein. The amide I band did not show any change in its position in the spectrum of GM; however, there is remarkable decrease in its intensity which could be attributed to denaturation of proteins. The FTIR spectra of the liver tissues of control and polluted samples shown in Fig. 4b revealed that there is slight blueshift of the amide I band with decrease in its intensity in polluted samples compared to the control, being more pronounced in GM sample. In the obtained FTIR spectra of the muscle samples of control and polluted fish shown in Fig. 4c, the amide I band did not show any change in its position in DM and Sh samples with only insignificant decrease in its intensity, while in GM sample, the amide I band demonstrated blueshift with remarkable decrease in its intensity in comparison with the control.

Additionally, there is a suggested change in the lipid profile in the gill samples of DM and Sh, manifested by a very obvious increase in the intensity of the stretching vibrations of CH of lipids at 2922 and 2852 cm^−1^, in addition to the emergence of new bands at ~ 3009 and 1743 cm^-1^ corresponding to vibrations of *Cis*-double bond of C-H stretching and C=O stretching from fatty ester group, respectively^58,62,63^, respectively. The liver sample of GM also demonstrated sharper and slightly more intense peaks of CH stretching vibrations in comparison to the control, with also emergence of additional bands at 3014, 1742 and 1709 cm^−1^ corresponding to *Cis*-double bond of C–H stretching, and C=O stretching from fatty ester and free fatty acid carboxylate^58,62,63^, respectively. Similar behavior in CH stretching bands was also observed in the spectra of muscles samples from DM and GM where the bands became slightly sharper and slightly more intense as compared to the control, while they completely disappeared in the FTIR spectrum of muscle sample from Sh. New bands representing C=O stretching from fatty ester also appears in the spectra of muscle samples from DM and GM at 1744 and 1743 cm^-1^, respectively.

**Fig. 5:**
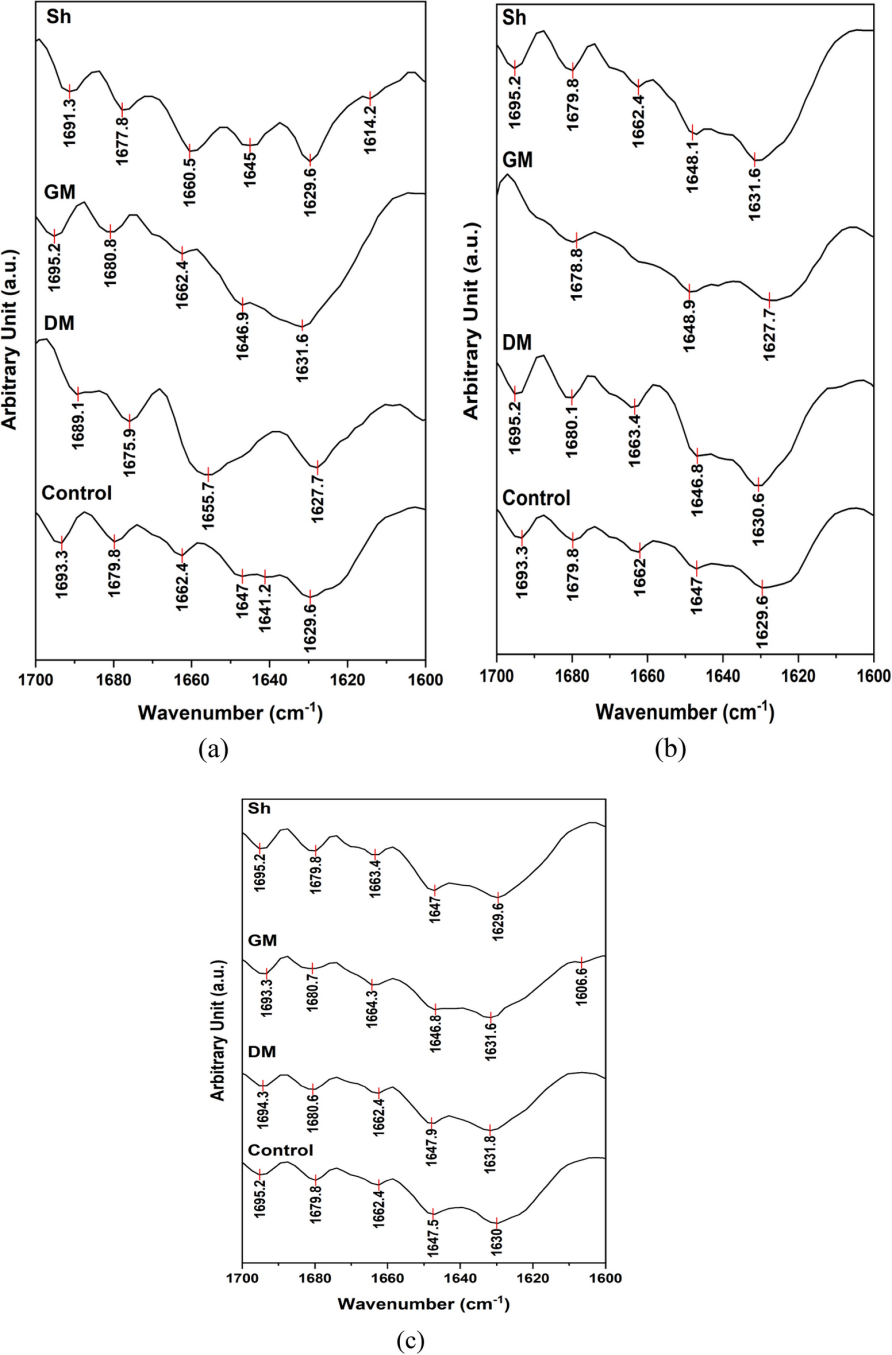
Second derivative spectra of the amide I region of (**a**) gills; (**b**) liver; and (**c**) muscles of control and polluted fish samples.

Since amide I band is sensitive to conformational changes in the secondary structures of proteins, it is often further analyzed to predict protein secondary structures^64^. Studying the second derivative of amide I is considered the best method in separating the its overlapped components thus revealing the secondary structure of protein and any changes in its conformations^65^. Figure 5 demonstrates the second derivative spectra of gills, liver and muscles of control and polluted fish samples. The detailed secondary structures band assignments for gills, liver and muscles are demonstrated in Tables 8, 9 and 10, respectively. The assigned secondary structures are as reported elsewhere^66–69^. Analysis of the secondary structures demonstrated the dominance of β-sheet and β-turn conformations in all the samples at expense α-helical conformations which were completely absence in the spectra of DM gill sample, DM and GM liver samples, and GM and Sh muscle samples. α-helical conformations are mainly stabilized by hydrogen bonds between carbonyl and amino groups, in addition to the electrostatic interactions between amino acids, and, consequently, changes to those two stabilizing factors could result in the loss of the α-helix structure^70^. In the current study, the decrease, or complete absence, of α-helix component associated with the persistence of β-sheet, random coil and β-turn conformations may, therefore, be attributed to the suggested alteration of the intermolecular hydrogen bonds resulting in partial protein unfolding^71,72^.

**Table 8:** Protein secondary structures of Control, DM, GM and Sh gill samples based on second derivative.

Sample	Wavenumber (cm^−1^)	Secondary structure
Control	1629.6, 1641.2, 1693.3	β-sheets
	1662.4	α-helix
	1679.8	β-turns
	1647	Random
DM	1627.7	β-sheets
	1675.9, 1689.1	β-turns
	1655.7	Random
GM	1631.6, 1695.2	β-sheets
	1662.4	α-helix
	1680.8	β-turns
	1646.9	Random
Sh	1614.2	Aggregate β-strands
	1629.6, 1691.3	β-sheets
	1660.5	α-helix
	1677.8	β-turns
	1645	Random

**Table 9:** Protein secondary structures of Control, DM, GM and Sh liver samples based on second derivative.

Sample	Wavenumber (cm^−1^)	Secondary structure
Control	1629.6, 1693.3	β-sheets
	1662	α-helix
	1679.8	β-turns
	1647	Random
DM	1630.6, 1695.2	β-sheets
	1663.4, 1680.1	β-turns
	1646.8	Random
GM	1627.7	β-sheets
	1678.8	β-turns
	1648.9	Random
Sh	1631.6, 1695.2	β-sheets
	1662.4	α-helix
	1679.8	β-turns
	1648.1	Random

**Table 10:** Protein secondary structures of Control, DM, GM and Sh muscles samples based on second derivative.

Sample	Wavenumber (cm^−1^)	Secondary structure
Control	1630, 1695.2	β-sheets
	1662.4	α-helix
	1680.6	β-turns
	1647.5	Random
DM	1631.8, 1694.3	β-sheets
	1662.4	α-helix
	1680.6	β-turns
	1647.9	Random
GM	1606.6	Aggregate β-strands
	1631.6, 1693.3	β-sheets
	1664.6, 1680.7	β-turns
	1646.8	Random
Sh	1626.6, 1695.2	β-sheets
	1663.4, 1679.8	β-turns
	1647	Random

### Quantitative bacteriological analyses

Water and soil pollution are considered to be one of the most dangerous hazards affecting not only in Egypt but also in the majority of world countries. The spoilage of water quality and water’s natural balance in its environment is known as water pollution^73^. Sediment is important reservoirs of microorganisms like pathogenic bacteria and exhibits a potential health hazards from possible re-suspension and subsequent ingestion during recreational activities^74^. Table 11 provides bacterial quality of the four different sites in water and sediment. The highest value of *S. aureus* (1.5 × 10^4^ CFU/ml) was found in Al-max water, while the lowest value (2.0 × 10^3^ CFU/ml) was recorded in El-Shatby water. Also, the same trend for the highest value was noticed in *E. coli* which was 5.0 × 10^3^ CFU/g in El-Max sediment. The lowest values were 1.0 × 10^3^ CFU/ml El-Shatby water. *Salmonella* spp*.* recorded the highest numbers (1.7 × 10^3^ CFU/ml) in El-Max water, while it is not detected in sediments of all sites (Table 11).

**Table 11:** Bacteriological characterization of water and sediment samples collected from different sites in Alexandria of Egypt.

Type of samples	Sampling sites	Foodborne pathogens Mean Plate Count CFU/ml (10^3^) ± SD		
		*E. coli*	*Salmonella* spp*.*	*S. aureus*
Water	Site 1	1.0 ± 1.2	ND	2.0 ± 0.01
	Site 2	ND	ND	ND
	Site 3	1.2 ± 0.3	0.2 ± 0.15	5.0 ± 0.11
	Site 4	3.3 ± 1.6	1.7 ± 0.32	15.0 ± 0.9
Sediment	Site 1	ND	ND	ND
	Site 2	ND	ND	ND
	Site 3	ND	ND	4.0 ± 0.8
	Site 4	5.0 ± 0.7	ND	7.0 ± 0.18

*ND* not detected.

### Bacterial analysis of fish samples

Marine fish are susceptible to a wide variety of bacterial pathogens. In the present study, concerning *Escherichia* spp. no colony was isolated from our malak and grouper fish samples. On the other hand, *Escherichia* spp. was detected in all fish samples (Table 12). The incidence of *S. aureus* isolated from gills are higher than liver in all fish species samples.

**Table 12:** Bacteriological characterization of fish samples collected from control site in Alexandria of Egypt.

Fish species	Fish Part	Foodborne pathogens Mean Plate Count CFU/g (10^3^) ± SD		
		*E. coli*	*Salmonella* spp*.*	*S. aureus*
Tilapia Fish	Liver	1.0 ± 0.11	4.8 ± 0.15	5.0 ± 0.13
	Gills	4.0 ± 0.20	6.0 ± 0.20	7.0 ± 0.04
	Muscles	ND	ND	ND
Red porgy Fish	Liver	1.0 ± 0.37	0.5 ± 0.18	1.0 ± 0.14
	Gills	1.0 ± 0.28	3.0 ± 0.36	5.0 ± 0.17
	Muscles	ND	ND	ND
Seabream Fish	Liver	1.0 ± 0.09	0.2 ± 0.13	6.0 ± 0.1
	Gills	1.0 ± 0.2	5.0 ± 0.27	8.0 ± 0.12
	Muscles	ND	ND	ND
Grouper Fish	Liver	ND	2.9 ± 0.06	1.0 ± 0.2
	Gills	ND	4.0 ± 0.09	3.0 ± 0.03
	Muscles	ND	1.0 ± 0.08	ND
Mullet Fish	Liver	2.0 ± 0.04	ND	1.5 ± 0.01
	Gills	4.0 ± 0.05	6.5 ± 0.5	5.0 ± 0.33
	Muscles	ND	ND	2.0 ± 0.43
Malak Fish	Liver	ND	ND	2.1 ± 0.29
	Gills	ND	2.8 ± 0.19	5.0 ± 0.23
	Muscles	ND	ND	ND

*ND* not detected.

The mean total counts of *E. coli*, *Salmonella* spp*.* and *S. aureus* in fish samples from polluted sites are listed in Table 13. The mean count of *Salmonella* spp. for fish samples was higher than the MPL defined by Egyptian standards^75^. This impact of bacterial load of fish will be more during summer season where ambient temperature would be higher than 30°C. Fish could be contaminated post-capture from fishing tools, contaminated water, contaminated ice, soiled surfaces and boxes, as well as by poor hygienic handling practices^76^. Environmental factors such as ambient temperature and relative humidity may play a role in the contamination of tilapia and the level of contamination could vary seasonally. Studies reported that, Gram-positive bacteria such as *Staphylococcus* spp. were increased in sea foods during storage at 2–4 °C. While storing fish at 0 °C as soon as possible after capturing will maintain the quality of fish by delaying the spoilage process^77^. Pollutants would increase the spoilage of fish and decreasing the shelf life. Bacterial pollutants not only affect the quality of fish but also may food safety problems^78^.

**Table 13:** Bacteriological characterization of fish samples collected from polluted site in Alexandria of Egypt.

Fish species	Fish Part	Foodborne pathogens Mean Plate Count CFU/g (10^3^) ± SD			Egyptian standard CFU/g*		
		*E. coli*	*Salmonella* spp.	*S. aureus*	*E. coli* (10^3^cfu/g)	*Salmonella* spp.**	*S. aureus* (10^3^ cfu/g)
Tilapia Fish	Liver	ND	1.0 ± 1.1	3.0 ± 1.9	100	0.0	1000
	Gills	10.0 ± 0.59	2.0 ± 0.84	5.0 ± 0.13			
	Muscles	ND	ND	ND			
Red porgy Fish	Liver	3.0 ± 0.45	1.4 ± 0.14	5.0 ± 0.39			
	Gills	12.0 ± 0.7	15.0 ± 0.7	10.0 ± 1.5			
	Muscles	2.0 ± 0.83	0.1 ± 0.28	3.0 ± 1.8			
Seabream Fish	Liver	1.0 ± 0.11	0.2 ± 0.35	7.0 ± 0.73			
	Gills	3.0 ± 1.74	18.0 ± 4.7	5.0 ± 0.78			
	Muscles	ND	ND	2.5 ± 1.6			
Grouper Fish	Liver	7.0 ± 0.67	1.3 ± 0.55	5.0 ± 0.35			
	Gills	12.0 ± 0.07	3.9 ± 0.61	7.0 ± 3.6			
	Muscles	1.0 ± 3.01	ND	0.2 ± 0.15			

ND: not detected.

*MPL stated by EOS (EOS, 2005) for fresh chilled fish, ** The count for *Salmonella* spp. Was not presented as enrichment steps were used.

### Micronucleus analysis

**Fig. 6:**
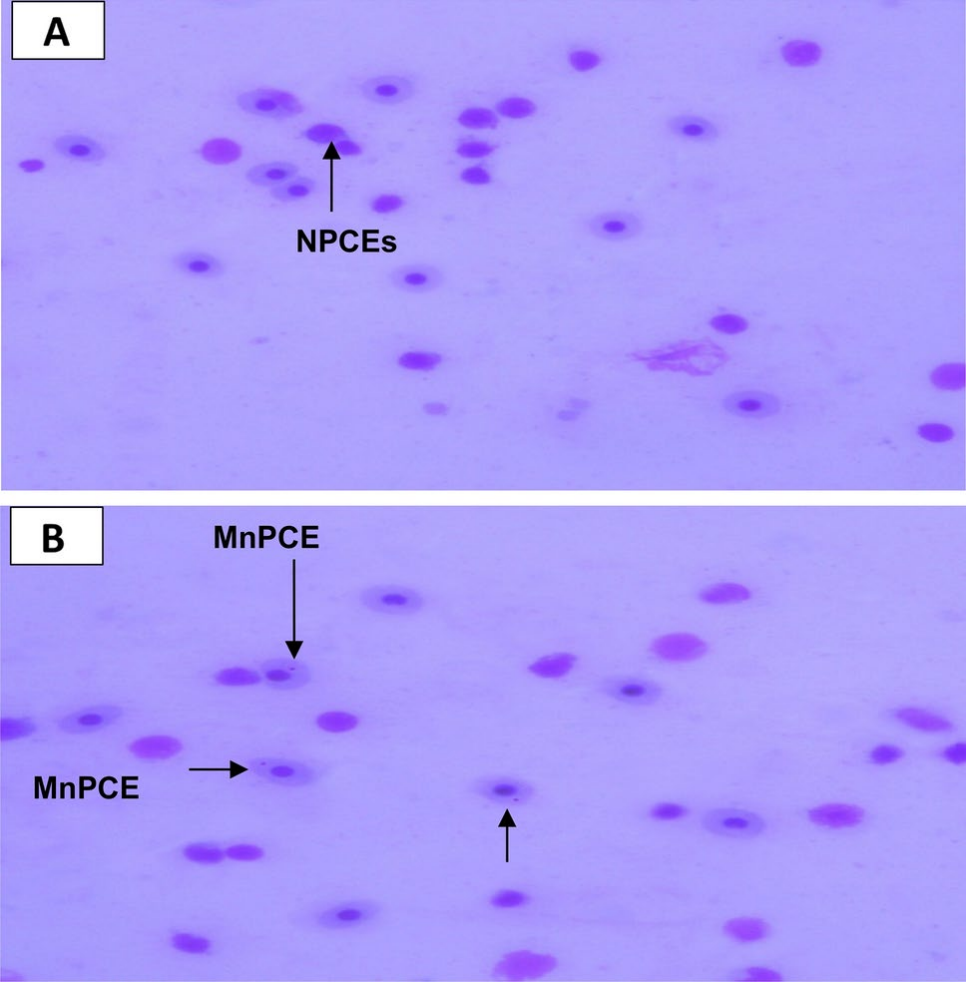
Micronucleated polychromatic erythrocytes (MnPCEs) from fish collected from: A: El-Shatby and B: El-Max.

The exposure of fish samples to different levels of pollution in Alexandria, Mediterranean Sea revealed differences in micronuclei (MN) between the highly polluted area (El-Max) and the less polluted (El-Shatby), Table 14 and Fig. 6. A significant increase in MnPCEs in micronuclei frequency detected in fish sampled from El-Max which receives pollution from different sources. However, no variation observed in fish collected from El-Shatby.

**Table 14:** Micronucleated polychromatic erythrocytes (MnPCEs) of fish collected from several location of Mediterranean Sea (mean ± SEM).

Location	MnPCEs/3000 PCEs
Control area	11.6 ± 0.4^d^
Site 1	25.2 ± 1.3^b^
Site 2	20.7 ± 0.8^c^
Site 3	29.4 ± 1.6^a^
Site 4	32.7 ± 2.3^a^

^a,b,c,d^Mean values within columns with unlike superscript letters were significantly different.

(*P* < 0.05).

The present study noticed that exposure of fish pollution in specific sites receiving different pollutants such as El-Maxrevealed a significant increase in micronuclei (MN) compared with fish collected from less polluted sites. El-Max receives different pollutants affecting fish health greatly^79^. Pollution highly affects fish samples and reflected in high numbers of MN which observed in a study by Abdellatif et al.^80^. Moreover, Malla et al.^81^ stated that nuclear abnormalities were identified in catfish as a result to exposure to genotoxic agents. Erythrocyte count is known as one of the first analyses to change in a stressful environment. In this research, it was noted that MN increased after the fish were moved from the control site to a polluted one.

### Gene expression analysis

**Fig. 7:**
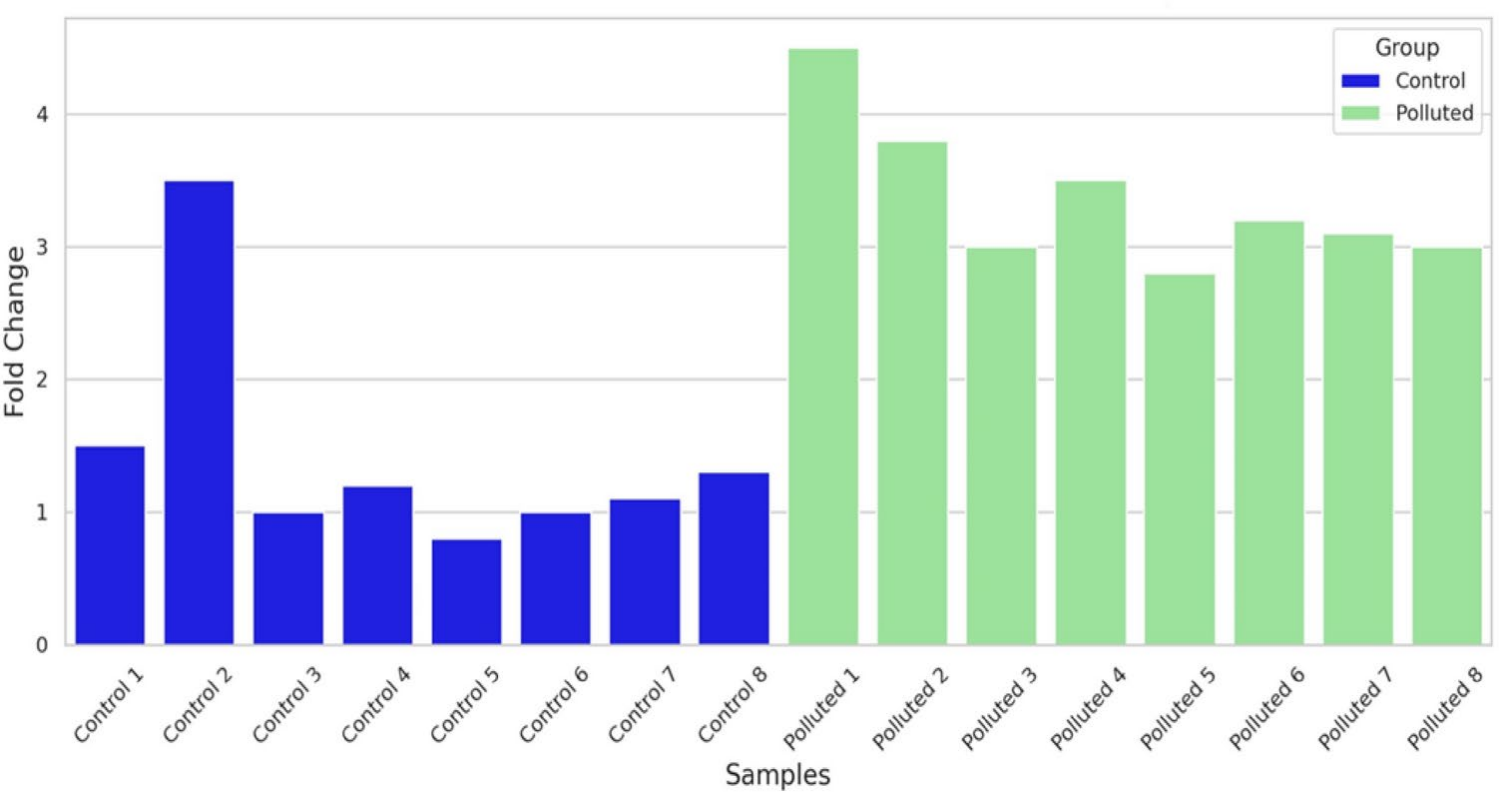
The relative expression of Cytochrome c in fish isolated from different sites, Alexandria (1, 2: tilapia gills and liver, 3, 4: Red porgy gills and liver, 5, 6: Sea bream and 7, 8: Grouper).

**Fig. 8:**
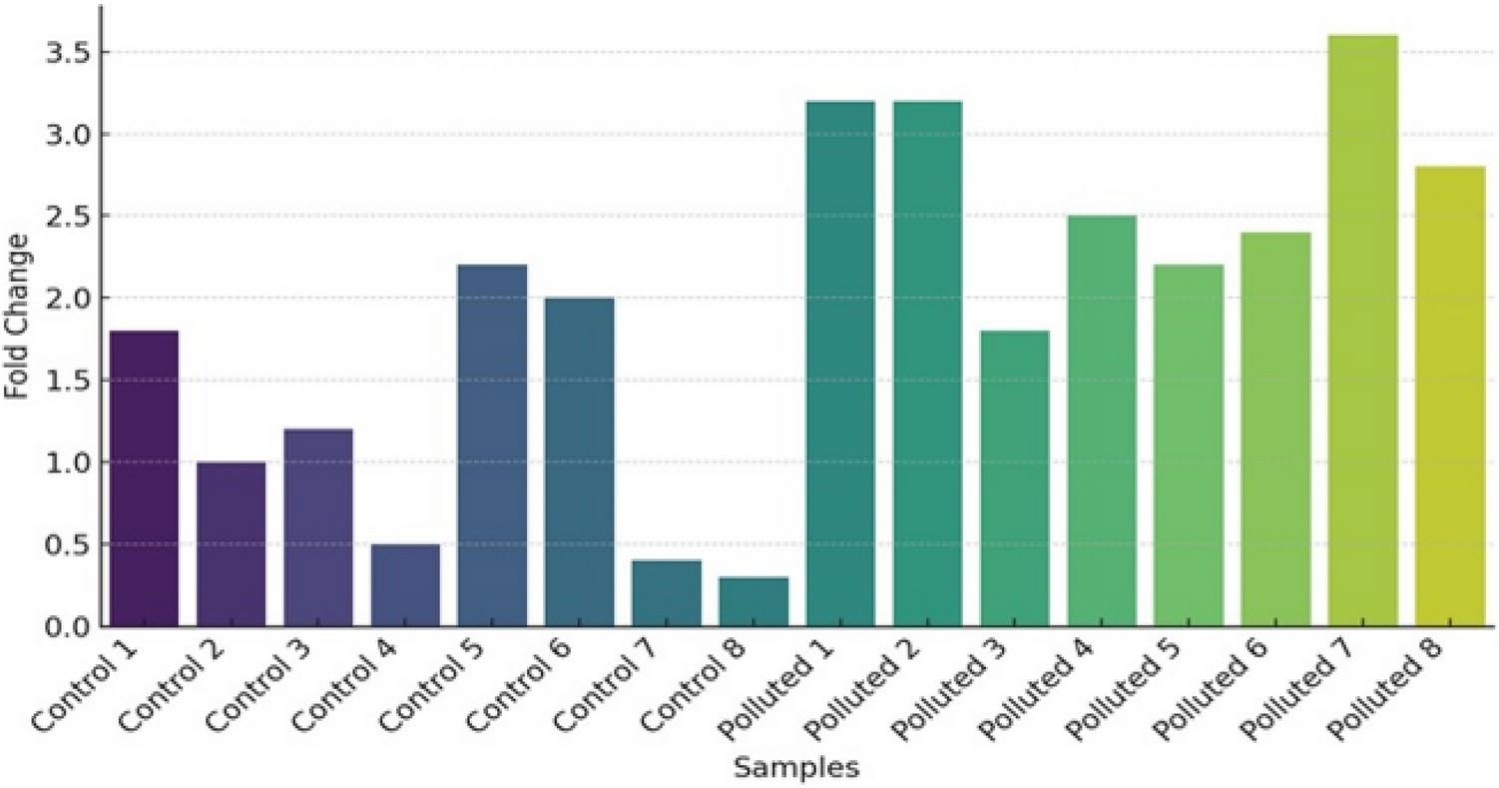
The relative expression of P53 in fish isolated from different sites, Alexandria. (1, 2: tilapia gills and liver, 3,4: Red porgy gills and liver, 5, 6: Sea bream gills and liver and 7, 8: Grouper gills and liver). One-way ANOVA indicates a statistically significant difference between control and polluted groups (*p* = 0.0001), suggesting a potential impact of pollution on gene expression levels in the studied fish species.

**Fig. 9:**
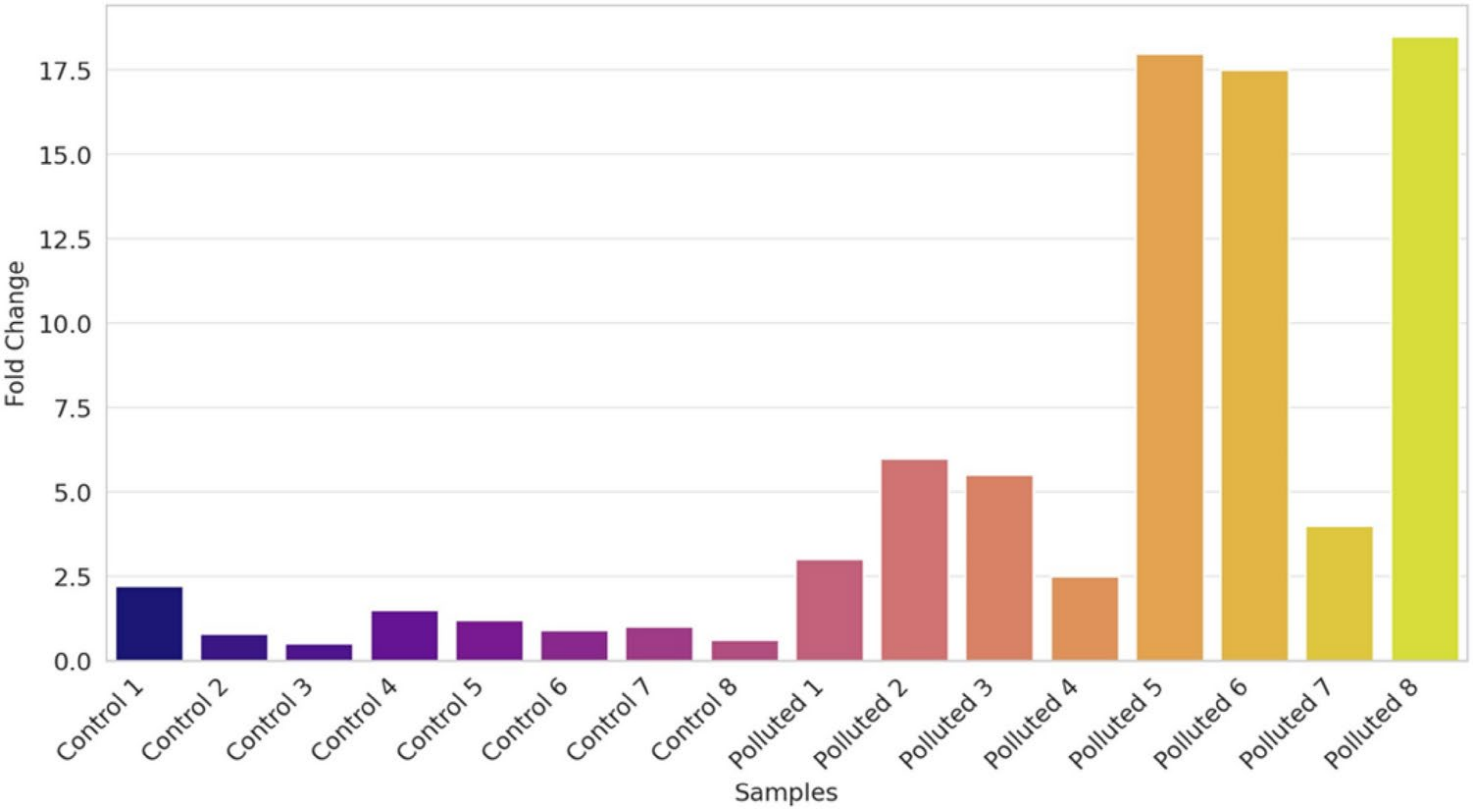
The relative expression of TNF in fish isolated from different sites, Alexandria. (1, 2: tilapia gills and liver, 3,4: Red porgy gills and liver, 5, 6: Sea bream gills and liver and 7, 8: Grouper gills and liver). A one-way ANOVA was performed to compare the fold change values between control and polluted groups. The results showed a statistically significant difference between the groups (F(1, 14) = 13.09, *p* = 0.0028), indicating that pollution has a significant effect on gene expression levels.

Gene expression of 3 important genes (Cytochrome c, P53 and TNF**)** displaying the impact of pollution on different fish samples was carried out in this research. Gills and liver were analyzed from four fish species: Tilapia, red porgy, sea bream and grouper. Primers were selected according to used fish and the sequence is listed in Table2. Results of gene expression are represented in Figs. 7, 8 and 9. The expression levels of Cytochrome C are seen in Fig. 7 is up regulated in fish collected from polluted sites when compared to control or less polluted. The highest levels of expression can be seen in red porgy fish collected from El-Max. Generally, high levels of gene expression are seen in all fish collected from El-Max when compared to fish collected from El-Shatby. The highest levels of Cytochrome C expression are seen in tilapia liver collected from El-Max.

The aquatic environment receives different pollutant such as toxic metals causing damage to mitochondria^82–84^, which results from mitochondrial respiratory chain damage releasing Cytochrome c into the cytosol to cause apoptosis^85^. Cytochrome c oxidase has a critical function during respiration of aquatic organisms by transferring electrons contributing to ATP generation^86^. Indeed, it has been proved that a reduced level of COX (subunit of Cytochrome c) activity led to functional reduction in Na+–K+-ATPase capacity, a major factor responsible for neuronal death in the mammalian brain^87^. In a similar manner to our experiment, expression levels of Cytochrome c increased as a result to Cadmium pollution^88^.

The fold change data were analyzed using one-way analysis of variance (ANOVA) to compare differences between control and polluted groups. The results showed a statistically significant difference between the two groups (F = 47.83, *p* = 7.14 × 10⁻⁶). The mean ± standard deviation (SD) for the control group was 1.48 ± 0.84, while for the polluted group, it was 3.74 ± 0.39. These findings indicate that Cytochrome c expression was significantly elevated in fish collected from polluted sites compared to those from control sites (Fig. 7).

The relative expression reached high levels in fish collected from El-Max when compared to fish sampled from El-Shatby. The highest levels of P53 are seen in tilapia fish collected from El-Max followed by grouper liver (Fig. 8). While the expression levels of TNF reached maximum levels only in sea bream and grouper (Fig. 9).

Apoptosis indicates the active process of cell death monitored by apoptotic genes to preserve the homeostasis of the organism’s interior environment. Apoptosis is induced by environmental stressors to cope with stimulated imbalances. However, excessive apoptosis might cause organ injury and cancer in fish^89,90^. The tumor suppressor transcription factor p53 stimulates many pathways in various cell cycles, senescence, and energy metabolism. The p53 gene is a major participant in controlling cell apoptosis. It mediates apoptosis through activating pro-apoptotic genes such as casp3 and gadd45ba^91^. Matching with our results high levels of P53 expression were recorded referring to exposure to environmental stressors, for example an increase in adaptive expression of the p53 gene was found in response to PAH exposure^92^. In research investigating the toxicity of microplastics and PAH on the Asian sea bass *Lates calcarifer*^93^, the proinflammatory gene TNF and the apoptosis related gene, P53 showed similar behavior to our results.

The inflammatory responses act as a vital role in most of serious physiological processes such as; cell repair and healing injured tissues and protecting the organisms from pathogens invasion^94^. The external stress can induce maturation of cytokines such as TNF which is known as a pleiotropic pro-inflammatory cytokine that activates a series of cytokines linked to proinflammatory^95^. In the present study; different detected pollutants in El-Max affected selected fish and resulted in large release of TNF. Similar results were found in pufferfish: the nitrite pollution could induce up-regulation of the mRNA expression of IL 6, IL 12 baff and genes TNFα^96^.

## Conclusion

The amino acids Ala, acts as model molecule simulating the structures of proteins in fish gills and liver, and PAla simulating the structures of proteins in fish muscle, in addition to their interactions with PAHs and hydrated Zn were all studied using DFT quantum mechanical calculations at B3LYP/6-31G(d,p) level to model the effect of both trace metals and PAHs on fish. The molecular structure of protein is affectedby the interaction with pollutants, as revealed by the change in the band positions of the amide groups in the computed IR spectra, as a result of the change in bond distances and bond angles caused by the interaction. As these findings needed expeimental verifcation,. the effect of pollution on the aquatic environment was also carried out including its effect on water, sediment and fish samples collected from four different sites. Direct confirmation of the results of molecular modeling was provided by FTIR spectroscopy. FTIR spectroscopy of gills, liver and muscle tissues of fish samples from the four different sites. The obtained FTIR spectra revealed obvious changes in the positions and intensities of amide I band of fish protein which could be attributed to disruption of the intermolecular hydrogen bonds associated with denaturation of protein, probably due to the interaction with pollutants, which confirms molecular modeling results. FTIR spectra also demonstrated changes in the lipid profile of the polluted samples in comparison to control, which could also be attributed to the effect of pollution. Bacterial examination of water and sediment samples from four different sites in Alexandria revealed that the highest value of *S. aureus* was found in El-Max water, while the lowest value was recorded in El-Shatby water. Also, the same trend for the highest value was noticed in *E. coli* which was in El-Max sediment. The lowest values were recorded in El-Shatby water. *Escherichia* spp. was detected in all fish samples. The incidence of *S. aureus* isolated from gills are higher than liver in all fish species samples. Results of micronucleus analysis revealed wide variation between control and polluted sites. Results of gene expression of the selected genes, Cytochrome c, P53 and TNF revealed that fish collected from El-Max showed higher levels than others collected from El-Shatby (considered as a control site). Tilapia fish showed higher levels than other fish. Results of both Cytochrome c and P53 were more representative than TNF. This study provides a systematic methodology, encompassing integration between molecular modeling, FTIR spectroscopy, bacteriological analysis, and micronucleus and gene expression analyses to reveal the detrimental impact of PAHs and heavy metals on aquatic organisms. We aim in the future work to further consolidate the methodology by including a remediation approach for both PAHs and heavy metals to treat the aquatic environment, then evalute the impact of this approach not only from a physicochemical perspective but also in terms of gene exrpession.

## Data Availability

The data will be available upon request. Contact Medhat A. Ibrahim, Email: ma.khalek@nrc.sci.eg.
